# Modern Treatment of Prostatic Hypertrophy1Read before the Buffalo Medical Union, June 27, 1900.

**Published:** 1900-08

**Authors:** Herman Mynter

**Affiliations:** Buffalo, N. Y., 566 Delaware Avenue


### MODERN TREATMENT OF PROSTATIC HYPERTROPHY.[1]

By HERMAN MYNTER, M. D., Buffalo, N. Y.

AS WE always have the poor with us, so we always have, and will continue to have, with us people with prostatic enlargement. "The days of our years are three score years and ten and if by reason of strength they be four score years, yet is their strength labor and sorrow, for it is soon cut off and we fly away!" Their strength is labor from the inability to empty the bladder, sorrow from the pain, the straining, the sleeplessness, the cystitis and pyelonephritis, resulting from the prostatic hypertrophy and we fly away in misery by uremia. The treatment of this painful disease has long been a snare and a delusion to the average physician. Nothing seems easier and more appropriate than to advise the regular employment of the catheter, when the amount of residual urine becomes so large that the patients walk about with their bladders practically full and are only able to void the overflow. The use of the catheter, however, is in the majority of cases bound to be followed by infection of the bladder, even if we do not consider the difficulties of introducing catheters on account of the irregularities of the prostatic urethra from hypertrophied median and lateral lobes, and the dangers of wounds and false passages from metallic catheters. The result of this infection is that the patients get from the ashes into the fire,—the fire of cystitis, strangury, pain, sleeplessness, hemorrhoids, rectal prolapse, hernia, hemorrhages, and progressive weakness and, emaciation—till, at last, the stinking alkaline urine infects the pelvis of the kidneys and the kidneys themselves, and the dry, furred tongue, the muscular twitchings and the progressive coma show that death from uremia is approaching. Surely, gentlemen, the picture is a sad one to contemplate, for the patient, the family and the physician, and we must hail with gratitude any means by aid of which we may be able to prevent such an outcome.

The prostate develops at puberty and atrophies after castration, showing that it is more a sexual than a urinary organ. Pathologically it has much in common with a fibromyoma of the uterus, particularly when it becomes hypertrophied. Both consist of the same kind of tissues, develop late in life, are apt to atrophy by castration or ovariotomy, or by ligation in the one place of the internal iliac arteries, in the other of the uterine arteries; both may be the subject

1. Read before the Buffalo Medical Union, June 27, 1900.

of similar retrogressive pathological changes, and both may be enucleated bluntly with the finger after splitting the capsule. After the sixtieth year, often earlier, the prostate becomes enlarged to double or even triple of its original size, the hypertrophy being either uniform in the whole organ or affecting the middle lobe or the lateral lobes, or both. The so-called median lobe is the upper posterior part, above the ejaculatory ducts, and is continuous with the lateral lobes, but when enlarged it forms a projection into the bladder, causing the posterior wall of the vesico-urethral opening to become convex. The prostatic urethra is thereby changed both in length and direction in its upper part and micturition and the passage of sounds are interfered with.

The prostate consists of stroma and glandular tissue, the stroma, according to Gerrish, forming more than three-fourths of the bulk of the normal gland. It consists of connective tissue and smooth muscular fibers and forms a sort of capsule to the prostate. By its outer surface this capsule is in contact and blended with the vesico-rectal fascia and the superior layer of the triangular ligament, while from its inner surface a system of trabecula radiate toward the veru montanum and divide the gland into a number of triangular spaces which lodge the gandular tissue; the glands number thirty or forty and are of the tubular variety. The ejaculatory ducts, three-quarters of an inch long, are formed close to the base and pass downward and forward side by side between the middle and lateral lobes.

It will easily be seen that by enlargement of the lateral or median lobes, separately or altogether, great changes will take place in the caliber, length, direction and curve of the prostatic urethra, which seriously will interfere with micturition and the introduction of instruments. If the lateral lobes be enlarged, the transverse diameter of the prostatic urethra will be lessened and its length increased according to the amount of hypertrophy, in some cases as much as three and a half inches. If the growth be one-sided, the canal will be deflected from its regular course in a lateral direction, the deviation being concave toward the affected side. If the median lobe is particularly affected, the floor of the bladder is lifted up and forms a projection into the bladder, which may become pedunculated and act as a valve, preventing micturition altogether. The upper part of the prostatic urethra curves then around this projection in a forward convex direction, which may prevent the introduction of a metallic catheter, while a double curved Mercier's catheter generally can be introduced. Behind the protruding lobe, and in a less degree in front of it, we frequently find a deep pouch in the bladder containing residual urine. The bladder cannot empty itself completely; the usual funnel-shaped entrance into the urethra is lost and its place taken by this projecting middle lobe, lifting the neck of the bladder upward above the base. The bladder is affected in a different way. If the hypertrophy is confined to one or both lateral lobes, the symptoms are very similar to those of organic stricture and we are apt to find the bladder in a state of concentric hypertrophy, on account of the frequent straining efforts to expell the urine. If the median lobe is chiefly affected and forms a valve-like projection into the bladder, the symptoms are those of more or less perfect retention, the bladder wall is thinned and atrophied, particularly in the pouches behind and in front of the projection, vesical atony is present, with great amount of residual urine.

If both stroma and glandular tissue are increased in their normal proportion, we have a true hypertrophy with an even enlargement and frequently without any serious symptoms. The common form, however, is a hypertrophy of the stroma alone, chiefly of the connective tissue, which forms a fibrous hypertrophy with no tendency to contraction. The glandular tissue is rarely alone affected and the hypertrophy is then less constant, the glandular tissue gradually disappearing and being replaced by a fibrous growth of greater density. That the hypertrophy may appear in the shape of simple tumor formation and confined to no particular portion of the gland is well known. Their importance depend upon the obstruction to the micturition, they produce. No treatment beside surgical extirpation is of any avail in these tumor formations. The hypertrophies proper, however, may be influenced by different surgical procedures, the results being better in the cases in which the hypertrophy is limited to the connective tissue or in which this greatly preponderates. The *symptoms* depend upon the obstruction to the passage of urine and secondarily upon inflammation of the bladder and upper urinary tract, brought about principally by the use of dirty instruments. On account of the straining we meet complications with hemorrhoids, hernia, hemorrhage, stone, rectal prolapse, and the like. In regard to *diagnosis* it is of importance to decide the character of the hypertrophy, its seat, extent and the presence of a complicating inflammation of the bladder and kidneys and its degree. A soft elastic swelling, felt by rectal palpation, indicates an excess of glandular tissue; a hard, often irregular and nodulated, swelling a preponderance of connective and fibrous tissue.

In hypertrophy of both lateral lobes the catheter is generally introduced with comparative ease, but the prostatic urethra is lengthened.

In unilateral hypertrophy, the tip of the catheter is deflected to the opposite side of the hypertrophied lobe. In hypertrophy of the middle lobe it may be difficult or impossible to introduce a metallic catheter, without, at least, greatly depressing the outer end of the instrument, in order to have the top surmount the inner projection, and we meet a great amount of residual urine. A Mercier's catheter with double curve will often enter the bladder with surprising ease, where it has been impossible to introduce a metallic catheter. Cystoscopic examination is of importance in order to study the form of the projection. In regard to treatment, it may safely be stated that as the symptoms depend upon the obstruction to the passage of urine, no treatment will be of any permanent use unless it provides, in some way or other, for the removal of the obstruction. I shall not therefore waste your time in discussing the various remedies and methods which have been supposed to exert some influence upon this hypertrophy, such as ergot, icthyol, iodides, etc., a mechanical treatment by dilatation, rectal massage and injections, all of which, at best, only mitigate the early symptoms or an additional engorgement.

The purely surgical treatment may be used for the purpose of producing shrinkage of the gland, by ligating the internal iliac arteries, by castration or by vasectomy, or—for the purpose of removing the obstruction partly or altogether—by prostatotomy, including Bottini's operation, or by prostatectomy in a more general hypertrophy, when the patient's condition still allows such an operation, or lastly, where neither seems indicated, for the purpose of relieving the patient from his continued miseries by establishing a suprapubic fistula.

Castration, introduced by White, of Philadelphia, in 1893, has proved itself to be a reliable and curative operation. I have performed it in about a dozen cases and the patients have all gradually been improved and relieved of their urinary troubles. I have seen none of the sudden improvements mentioned by different authors. The improvement has in every case been slow and it has taken months to accomplish the shrinkage. The operation seems to have most effect in cases with hypertrophy of muscular and glandular tissue; in short, in cases in which the prostate feels soft and evenly swollen by rectal palpation. The mortality, however, is given at about 10 per cent, and improvement has been noted in about 94 per cent. of the cases who survived the operation. The power of the bladder returned in 66 per cent. with disappearance of the cystitis. The authors differ in regard to the mental disturbances frequently observed after castration in old men, some calling it acute mania, others traumatic delirium or uremia. None of my cases have suffered from it and none have died as the result of the castration.

*Vasectomy* has been recommended as a substitute for castration, on the supposition that it would result in atrophy of the testes and secondarily in atrophy of the prostate, and that without unsexing the patients. In my hands, this operation has had no effect upon the size of the prostate. Wood states that while the testes did not atrophy, a slowly progressive shrinkage of the prostate did take place. Reginald Harrison[1] considers vasectomy especially effectual in the early stages of prostatic enlargement by effecting shrinkage, but that prostatectomy is preferable in cases in which the prostate has assumed the form and structure of fibrous growth and the obstruction warrants other measures than catheterism. He believes that the shrinkage following vasectomy may do good in making it easier to introduce the catheter and in removing spasms, pain and hemorrhage, although he considers it impossible to restore the natural function of the bladder in cases in which secondary changes, such as sacs, pouches or trabeculation, have taken place in the bladder. I am free to state that I have seen no effect whatever of vasectomy upon the size of the prostate or upon the symptoms.

*Ligation* of the internal iliac arteries has been recommended and performed a few times by Willy Meyer, of New York, and apparently with good success. It is, however, a formidable operation, difficult to perform and not without great danger in old and feeble patients.

*Prostatotomy*, to which belongs Bottini's electro-caustic operation, is only indicated in hypertrophy of the middle lobe. It means the division of the projection or bar at the neck of the bladder by aid of special instruments introduced through the urethra or after external urethrotomy, and the operation is apt to be followed by severe hemorrhage and infection. The success of the operation is probably more due to the continuous drainage after the operation than to the operation itself. The operation is a makeshift, at best, and is only suited to those cases in which cystitis has gradually become acute and the passage of instruments difficult and in which no other operation is allowed. Occasionally, by sheer luck, it may be possible through the perineal wound to diagnosticate and remove a projecting, pedunculated median lobe and so give permanent relief.

Bottini's operation, introduced in 1875 and later, in 1897, greatly improved by Freudenberg, of Berlin, is not without danger and its

1. Medical Fortnightly (St. Louis), May 25, 1900.

usefulness is limited to cases with a median projection. Severe hemorrhage is apt to occur and has occurred, a large and expensive apparatus is required and it demands a manual dexterity which only exceptionally is found. The last statistics of 229 cases, shows 57.5 per cent. cures, 26 per cent. improvements, 13 per cent. without results and 8 per cent. deaths. In a number of cases, besides, the cures or improvements were not permanent.

The most modern operation is prostatectomy; the enucleation of the hyperthrophied middle and lateral lobes through a perineal incision, combined with, and preceded by, a suprapubic cystotomy, through which two fingers are introduced into the bladder and the prostate crowded down towards the perineum. This operation which bears Alexander's name, is performed in the following way: After preliminary suprapubic cystotomy a grooved stone sound is introduced into the bladder through the urethra, with the patient in the lithotomy position, the membranous urethra is incised by a median incision, reaching from the bulbus urethra to just in front of the anus and including the tip of the prostate. The staff is now removed and while the prostate is being crowded downward with two fingers in the bladder, the second finger of the right hand is introduced through the perineal incision, the fibrous sheath of the prostate is broken into by the finger, the capsule entered and the hypertrophied lobes enucleated bluntly with the finger. Drainage is thereafter established, both through the suprapubic and the perineal incision, for about a week. The suprapubic cystotomy may be necessary in certain patients with large abdomens, but is, of course, a serious complication in old people. Parker Syms[1] has therefore proposed to open the abdominal cavity above the symphysis and introduce the hand and press the prostate down through the intact bladder wall. His argument, of course, is that such a laparatomy with proper precautions is without danger.

Johnson[2] has modified this idea by simply making a suprapubic incision without opening either the bladder or the peritoneum and through this incision press the prostate downward. I believe that even this incision is unnecessary and that the operation can be performed without any suprapubic incision at all, as I in a recent case in a large and heavy man, during the anesthesia discovered that I could press the prostate downward with great force by bi-manual examination, one finger being in the rectum and the other hand pressing

1. Annals of Surgery, March, 1899.
2. Annals of Surgery, June, 1900.

down above the symphysis. I was able in that way to palpate the upper margin of the greatly enlarged prostate. To make the operation easier, and better to expose the prostate, I, having previously introduced a stone sound, let the posterior part of the median incision curve around the anterior half of the anus on both sides, dissected loose the whole anterior part of the ampulla of the rectum, exposed the prostate to sight, incised the capsule in the middle line, but without opening the urethra, and through this incision enucleated with ease both hypertrophied lateral and median lobes. The prostatic urethra was, however, torn during the process of enucleation and through this opening, I therefore, introduced a large drain and kept it there for seven days. The patient, a large fleshy man, 67 years of age, has so far been doing well. Before the operation he was forced to pass water day and night every hour and a half and had considerable cystitis. He was operated on June 15th and is today, July 8th, out of bed; can keep his water four hours, his cystitis is rapidly improving and I confidently expect a perfect recovery. The weight of the enucleated lobes were $2\frac{3}{4}$ ℥ or 88 grams, a weight that sufficiently shows the enormous size of the prostate. How far it is possible to perform this operation without tearing the urethra, I am not prepared to state. It was torn in my case and therefore I drained. I kept, however, a large grooved stone sound in the urethra during the operation and the urethra was torn inside the prostate by the contact of the finger with its rather sharp edge. I believe this accident can be avoided by using simply a Mercier's catheter, instead of a stone sound. It is firm and hard enough to locate the urethra with the finger inside the capsule of the prostate. One thing, however, I am prepared to state: that it is not necessary to make the suprapubic cystotomy which, at best, is a serious complication.

Alexander has twice performed this operation on very thin subjects without suprapubic cystotomy. My patient, however, was a large and fleshy man and the operation was, nevertheless, done with ease without cystotomy. He had two years previously been submitted to vasectomy, but without any benefit. He had arrived at the time when he was obliged to empty, or rather try to empty, his bladder every hour and a half night and day; he had considerable cystitis from using catheters and he was willing to submit to any operation that promised relief. I advised, therefore, a radical operation, a la Alexander, but avoiding the serious complication of supra pubic cystotomy. With a Mercier's catheter in the bladder instead of a grooved stone sound, I feel confident I could have avoided the tear in the urethra, and we would then have an ideal operation with the bladder and the urethra intact and a simple wound in the prostate, which would only keep the patient in bed for a couple of days and soon would heal by granulation.

My belief is that this modification of Alexander's operation will become the operation of preference in the future and be performed early, when the symptoms of obstruction commence to develop and before the bladder and the kidneys have become involved.

566 Delaware Avenue.

